# Probing clarity: AI-generated simplified breast imaging reports for enhanced patient comprehension powered by ChatGPT-4o

**DOI:** 10.1186/s41747-024-00526-1

**Published:** 2024-10-30

**Authors:** Roberto Maroncelli, Veronica Rizzo, Marcella Pasculli, Federica Cicciarelli, Massimo Macera, Francesca Galati, Carlo Catalano, Federica Pediconi

**Affiliations:** 1 Department of Radiological, Oncological and Pathological Sciences, Sapienza—University of Rome, Rome, Roma, Italy; 2 Federico II—University of Naples, Naples, Italy

**Keywords:** Artificial intelligence, Breast radiology, Large language models, Natural language processing, Patient-centered care

## Abstract

**Background:**

To assess the reliability and comprehensibility of breast radiology reports simplified by artificial intelligence using the large language model (LLM) ChatGPT-4o.

**Methods:**

A radiologist with 20 years’ experience selected 21 anonymized breast radiology reports, 7 mammography, 7 breast ultrasound, and 7 breast magnetic resonance imaging (MRI), categorized according to breast imaging reporting and data system (BI-RADS). These reports underwent simplification by prompting ChatGPT-4o with “Explain this medical report to a patient using simple language”. Five breast radiologists assessed the quality of these simplified reports for factual accuracy, completeness, and potential harm with a 5-point Likert scale from 1 (strongly agree) to 5 (strongly disagree). Another breast radiologist evaluated the text comprehension of five non-healthcare personnel readers using a 5-point Likert scale from 1 (excellent) to 5 (poor). Descriptive statistics, Cronbach’s α, and the Kruskal–Wallis test were used.

**Results:**

Mammography, ultrasound, and MRI showed high factual accuracy (median 2) and completeness (median 2) across radiologists, with low potential harm scores (median 5); no significant group differences (*p* ≥ 0.780), and high internal consistency (α > 0.80) were observed. Non-healthcare readers showed high comprehension (median 2 for mammography and MRI and 1 for ultrasound); no significant group differences across modalities (*p* = 0.368), and high internal consistency (α > 0.85) were observed. BI-RADS 0, 1, and 2 reports were accurately explained, while BI-RADS 3–6 reports were challenging.

**Conclusion:**

The model demonstrated reliability and clarity, offering promise for patients with diverse backgrounds. LLMs like ChatGPT-4o could simplify breast radiology reports, aid in communication, and enhance patient care.

**Relevance statement:**

Simplified breast radiology reports generated by ChatGPT-4o show potential in enhancing communication with patients, improving comprehension across varying educational backgrounds, and contributing to patient-centered care in radiology practice.

**Key Points:**

- AI simplifies complex breast imaging reports, enhancing patient understanding.
- Simplified reports from AI maintain accuracy, improving patient comprehension significantly.
- Implementing AI reports enhances patient engagement and communication in breast imaging.

**Graphical Abstract:**

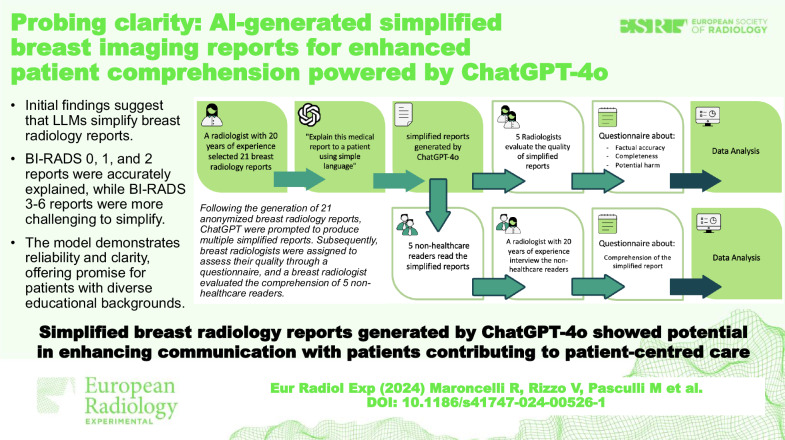

## Background

In the realm of diagnostic imaging, the advent of artificial intelligence (AI), particularly large language models (LLMs) like ChatGPT [1] and its last version ChatGPT-4o [2], marked a significant evolution toward enhancing radiological reporting and patient communications [3]. This transformation aligns with growing demands for patient-centered care, emphasizing the need for comprehensible medical reports [4]. Despite the progressive strides in radiological technology and patient communication strategies, a persistent challenge remains: the complexity of radiological reports often renders their meaning incomprehensible to patients lacking medical expertise [5]. Addressing this challenge is crucial, as it impacts patient understanding, satisfaction, and engagement in their healthcare processes [5, 6].

With patients increasingly informed and having free access to the Internet, there is a risk of misinterpreting radiological reports, which AI could either exacerbate or mitigate. Recent advancements in AI have introduced the potential to simplify complex medical terminologies into patient-friendly language, thereby bridging the gap between radiological evaluations and patient comprehension [3, 7].

This study used ChatGPT-4o’s capabilities [2] to compare original and simplified breast radiological reports, focusing on mammography, breast ultrasound, and breast magnetic resonance imaging (MRI) reports categorized according to the American College of Radiology breast imaging reporting and data system (BI-RADS) classification system (BI-RADS®) [8], evaluating their accuracy, completeness, and safety.

The primary objective was to investigate the practical utility of AI in the medical field and explore its potential to enhance patient-centered care. By integrating AI tools more widely into clinical practice, we aim to make radiological information accessible and helpful to all patients, regardless of their medical knowledge although this is not yet possible due to current regulations.

## Methods

This prospective investigation was founded upon randomly selected anonymized breast radiology reports; thus, no actual patient data was incorporated into this prompt. Before participation, written informed consent, regarding the use of the data, their anonymization, the purpose of the study, and the independence of the evaluations was acquired from the involved readers. Their responses were compiled through anonymized questionnaires. Adhering to the guidelines stipulated by the Institutional Review Board, ethical consultation was deemed unnecessary for the endorsement of this study.

**Fig. 1:**
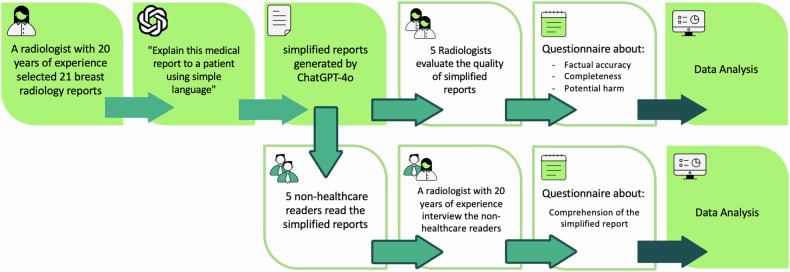
Flowchart, following the random selection of 21 anonymized breast radiology reports, ChatGPT was prompted to produce multiple simplified reports. Subsequently, five breast radiologists are assigned to assess their quality through a questionnaire, and a breast radiologist evaluates the comprehension of five non-healthcare readers (NHRs)

To evaluate radiology reports streamlined through ChatGPT-4o, we followed the protocol summarized in the flowchart shown in Fig. 1.

### Original radiology reports

A breast radiologist with 20 years of experience (F.P.) randomly selected from her archive, 21 anonymized breast radiology reports, 7 for each evaluated imaging modality: mammography, breast ultrasound. and breast MRI. Each report was categorized according to the BI-RADS classification system [8], providing a diverse range of cases for evaluation, with at least one report for each diagnostic category, from 0 to 6, for every imaging modality. The reports, questionnaires, and interviews were conducted in Italian, the original language of the reports and the native language of all subjects involved in the study. Table 1 summarizes the given name and the BI-RADS category of each report.

**Table 1:** Given name and ACR BI-RADS category of each report

Mammography report	BI-RADS category
1a	0
2a	1
3a	2
4a	3
5a	4
6a	5
7a	6

Ultrasound report	BI-RADS category
1b	0
2b	1
3b	2
4b	3
5b	4
6b	5
7b	6

MRI report	BI-RADS category
1c	0
2c	1
3c	2
4c	3
5c	4
6c	5
7c	6

### AI simplification process

The initial radiology reports were simplified by activating the ChatGPT-4o web interface on May 20th, 2024, with the inquiry “Explain this medical report to a patient using simple language” followed by the unaltered findings presented in plain text. This inquiry was based on a heuristic analysis of different reports and was reviewed by the same breast radiologist with 20 years of experience, who selected the reports and deemed them the most effective for producing simplified summaries.

The ChatGPT-4o interface version utilized in this research lacked the option to modify any model configurations, resulting in outputs with unpredictable attributes [1, 2]. To accommodate for this variability in outputs and ensure a comprehensive exploration of its generative potential, we initiated the model and presented the inquiry ten times for each of the 21 original reports. This process yielded 210 distinct simplified renditions of the reports.

**Fig. 2:**
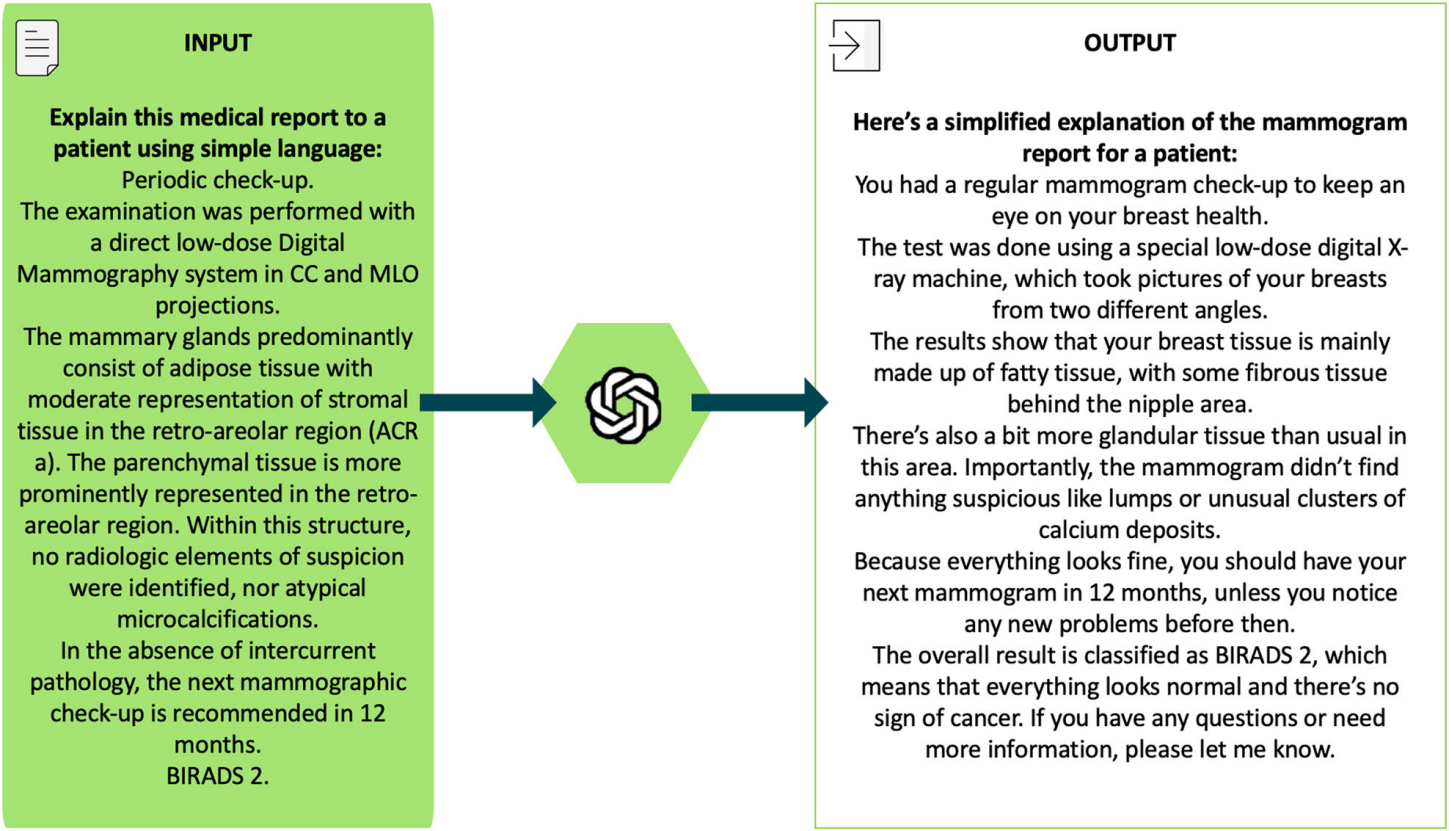
Example of the simplification process of a mammography report with ChatGPT-4o

An example of the simplification process of a report with ChatGPT-4o is available in Fig. 2.

### Questionnaire and evaluation procedure

**Fig. 3:**
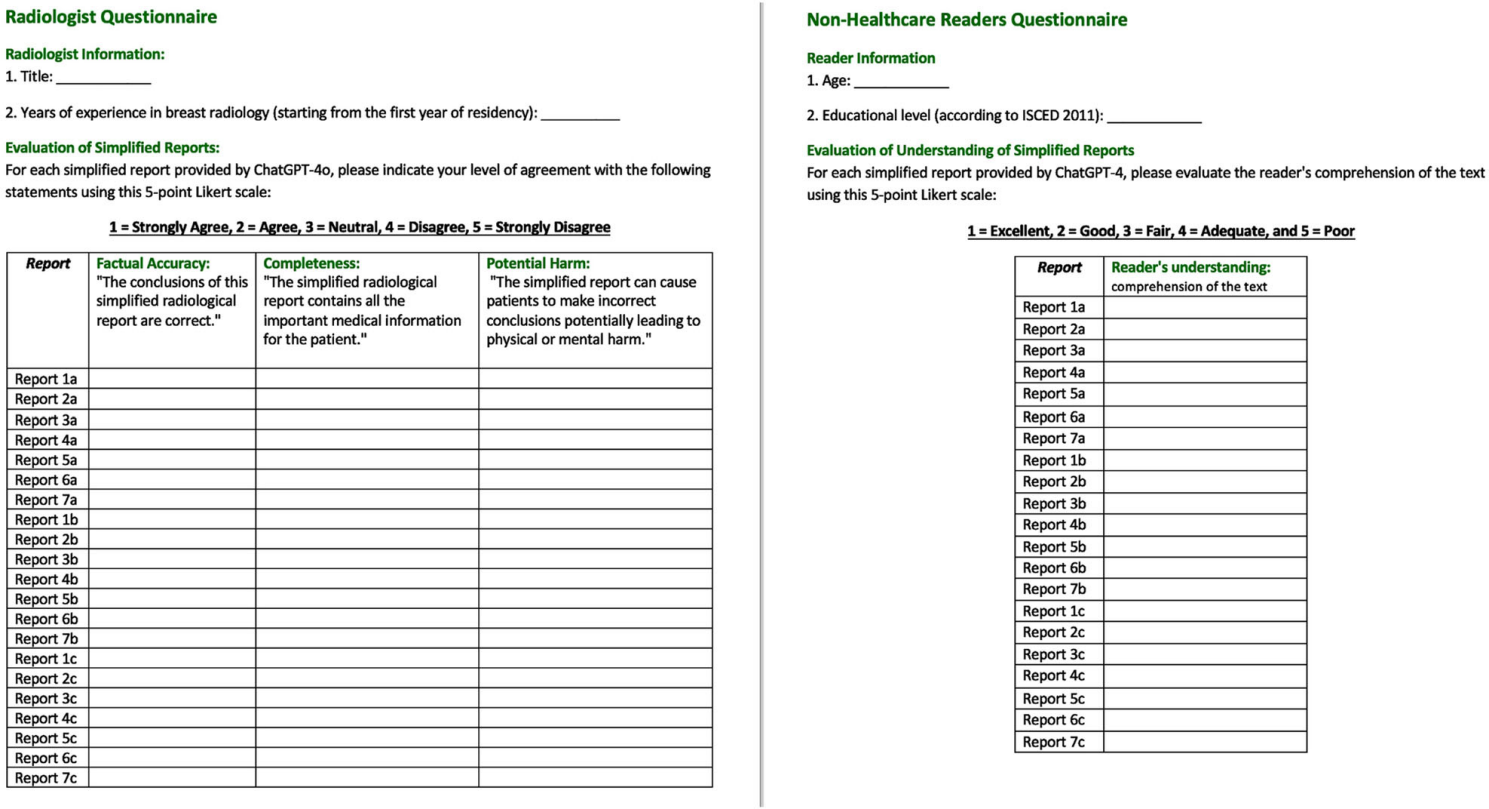
The questionnaires designed for the Radiologist’s evaluation (on the right) and for the understanding of NHRs (on the left)

Five breast radiologists from our hospital, each with different levels of experience in terms of academic title and years of practice in breast radiology (Table 2), were tasked with independently evaluating the quality of simplified reports generated by ChatGPT-4o through a questionnaire across three parameters: factual accuracy; completeness; and potential harm) (Fig. 3).

**Table 2:** Characteristics of the readers

Breast radiologist readers (BRRs)	Position (academic title)	Years of experience in breast imaging
#1	Resident	2
#2	Resident	4
#3	Ph.D.	10
#4	Ph.D.	15
#5	Prof.	20

Non-healthcare reader (NHRs)	Age (years)	Educational level (ISCED 2011)
#1	55	3
#2	24	4
#3	31	5
#4	65	6
#5	70	7

*ISCED* International Standard Classification of Education [9]

Before the assessment, each breast radiologist reader (BRR) was briefed on the origin of the simplified radiology reports, clarifying that they were generated using the LLM ChatGPT-4o. BRRs were informed about the purpose of the study and given detailed instructions on questionnaire completion explaining how to use the 5-point Likert scale.

Additionally, BBRs were asked to specify their years of experience in breast radiology starting from the first year of residency. Each questionnaire included the 21 original reports, and, for each report, a randomly selected, unique simplified version generated by ChatGPT-4o. Subsequently, participants were presented with a series of questions designed to gauge the quality of the simplified reports (Table 3).

**Table 3:** Questions used to assess the quality of radiology reports simplified with ChatGPT

Quality category	Likert scale statements
Factual accuracy	The conclusions of this simplified radiological report are correct.
Completeness	The simplified radiology report contains all the important medical information for the patient.
Potential harm	The simplified report can cause patients to make incorrect conclusions, potentially leading to physical or mental harm.

Likert scale statements were answered on a 5-point scale (1 = strongly agree; 2 = agree; 3 = neutral; 4 = disagree; and 5 = strongly disagree)

The BRRs assessed factual accuracy, completeness, and potential harm for each of the 21 cases. For each of the three quality aspects, respondents were required to indicate their level of agreement with corresponding statements using a 5-point Likert scale (score 1 = strongly agree; score 2 = agree; score 3 = neutral; score 4 = disagree; and score 5 = strongly disagree). The questionnaires were collected and checked for consent and completeness by a breast radiologist (R.M.) in charge of administering the written questionnaire.

In addition, five readers, none being healthcare personnel (non-healthcare readers [NHRs]), simulating patients with different levels of education without specific training in medicine, were selected according to the International Standard Classification of Education (ISCED) 2011 criteria [9], with a minimum level of 3 (Table 2). They were provided with a randomly selected, unique simplified version generated by ChatGPT-4o of the 21 reports. Following this, NHRs underwent questioning by the breast radiologist with 20 years of experience (F.P.), who assessed their comprehension of the text. Subsequently, this radiologist graded the NHRs’ understanding using a 5-point Likert scale (1 = excellent; 2 = good; 3 = fair; 4 = adequate; and 5 = poor) assessing the comprehension of the final message and recommendations in the report through a questionnaire (Fig. 3).

### Statistical analysis

We performed a descriptive analysis calculating the statistical parameters for the ordinal scales: median, 25th percentile, 75th percentile, interquartile range (IQR), minimum, and maximum both for the evaluations given by the BRBs and for those given by the NHRs.

To assess the reliability of BRRs’ and NHRs’ assessments, we performed an internal consistency test (Cronbach’s α). To compare the distributions of the ratings between the different radiologists and evaluate whether there were significant differences, we used the non-parametric Kruskal–Wallis test.

To assess the difference between the BI-RADS 0, 1, and 2 reports *versus* BI-RADS 3, 4, 5, and 6 reports and BI-RADS 3 *versus* BI-RADS 4 and 5, using the Mann–Whitney *U*-test.

The statistical significance level was set at *p* < 0.05 for all tests, and we used Microsoft Excel, version 16.88, and IBM SPSS Statistics software, version 28, for all statistical analyses and graph generation.

## Results

### BRRs’ assessments

#### Descriptive analysis of the BRRs’ scores for mammography, ultrasound, and MRI reports

**Fig. 4:**
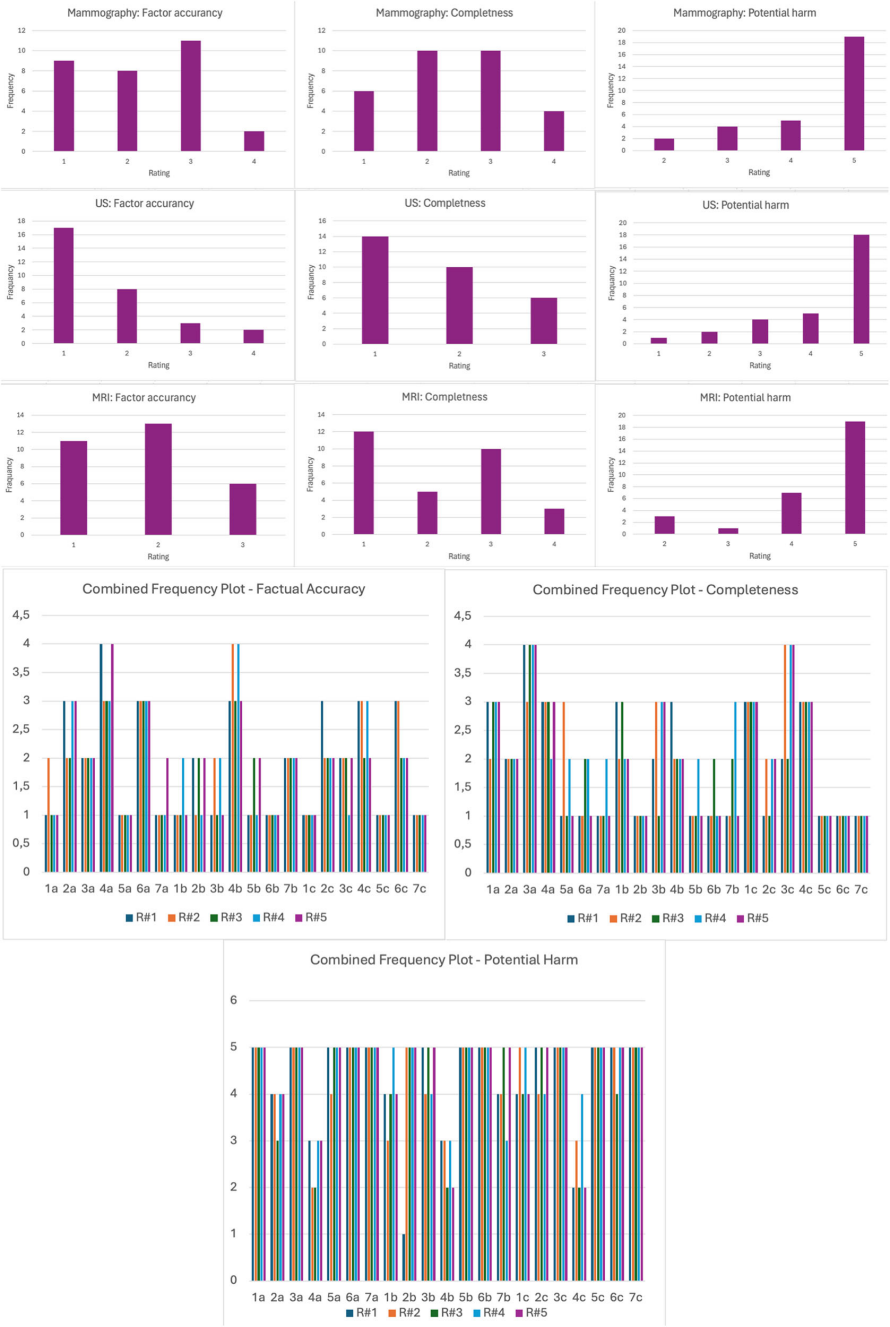
The frequency of the radiologists’ ratings for mammography, ultrasound (US), and MRI reports (on the top), as well as the combined frequency for factual accuracy, completeness, and potential harm (on the bottom), using a 5-point Likert scale (1 = strongly agree; 2 = agree; 3 = neutral; 4 = disagree; and 5 = strongly disagree)

The results for each BRR rating across the three evaluation criteria and the three different imaging modalities, including the combined analysis, are detailed in Table 4. Frequency graphs of the BRRs’ ratings are presented in Fig. 4.

**Table 4:** Summary statistics for the three categories factual accuracy, completeness, and potential harm

Criterion	Modality	Radiologist	Count	Mean	Median	SD	Min	Q1	IQR	Q3	Max
Factual accuracy	Mammography	#1	7	2.14	2	1.21	1	1.0	2	3.0	4
		#2	7	2.00	2	0.82	1	1.5	2	2.5	3
		#3	7	1.86	2	0.90	1	1.0	2	2.5	3
		#4	7	2.00	2	1.00	1	1.0	2	3.0	3
		#5	7	2.29	2	1.11	1	1.5	2	3.0	4
	Ultrasound	#1	7	1.57	1	0.79	1	1.0	1	2.0	3
		#2	7	1.71	1	1.11	1	1.0	1	2.0	4
		#3	7	1.71	2	0.76	1	1.0	2	2.0	3
		#4	7	1.86	2	1.07	1	1.0	2	2.0	4
		#5	7	1.71	2	0.76	1	1.0	2	2.0	3
	MRI	#1	7	2.00	2	1.00	1	1.0	2	3.0	3
		#2	7	1.86	2	0.90	1	1.0	2	2.5	3
		#3	7	1.57	2	0.53	1	1.0	2	2.0	2
		#4	7	1.57	1	0.79	1	1.0	1	2.0	3
		#5	7	1.57	2	0.53	1	1.0	2	2.0	2
	Combined	#1	21	1.90	2	1.00	1	1.0	2	3.0	4
		#2	21	1.86	2	0.91	1	1.0	2	2.0	4
		#3	21	1.71	2	0.72	1	1.0	2	2.0	3
		#4	21	1.81	2	0.93	1	1.0	2	2.0	4
		#5	21	1.86	2	0.85	1	1.0	2	2.0	4
Completeness	Mammography	#1	7	2.14	2	1.21	1	1.0	2	3.0	4
		#2	7	2.14	2	0.90	1	1.5	2	3.0	3
		#3	7	2.29	2	1.11	1	1.5	2	3.0	4
		#4	7	2.43	2	0.79	2	2.0	2	2.5	4
		#5	7	2.14	2	1.21	1	1.0	2	3.0	4
	Ultrasound	#1	7	1.71	1	0.95	1	1.0	1	2.5	3
		#2	7	1.57	1	0.79	1	1.0	1	2.0	3
		#3	7	1.71	2	0.76	1	1.0	2	2.0	3
		#4	7	2.00	2	0.82	1	1.5	2	2.5	3
		#5	7	1.57	1	0.79	1	1.0	1	2.0	3
	MRI	#1	7	1.71	1	0.95	1	1.0	1	2.5	3
		#2	7	2.14	2	1.21	1	1.0	2	3.0	4
		#3	7	1.71	1	0.95	1	1.0	1	2.5	3
		#4	7	2.14	2	1.21	1	1.0	2	3.0	4
		#5	7	2.14	2	1.21	1	1.0	2	3.0	4
	Combined	#1	21	1.86	1	1.01	1	1.0	1	3.0	4
		#2	21	1.95	2	0.97	1	1.0	2	3.0	4
		#3	21	1.90	2	0.94	1	1.0	2	3.0	4
		#4	21	2.19	2	0.93	1	2.0	2	3.0	4
		#5	21	1.95	2	1.07	1	1.0	2	3.0	4
Potential harm	Mammography	#1	7	4.57	5	0.79	3	4.5	5	5.0	5
		#2	7	4.29	5	1.11	2	4.0	5	5.0	5
		#3	7	4.29	5	1.25	2	4.0	5	5.0	5
		#4	7	4.57	5	0.79	3	4.5	5	5.0	5
		#5	7	4.57	5	0.79	3	4.5	5	5.0	5
	Ultrasound	#1	7	3.86	4	1.46	1	3.5	4	5.0	5
		#2	7	4.14	4	0.90	3	3.5	4	5.0	5
		#3	7	4.43	5	1.13	2	4.5	5	5.0	5
		#4	7	4.29	5	0.95	3	3.5	5	5.0	5
		#5	7	4.43	5	1.13	2	4.5	5	5.0	5
	MRI	#1	7	4.43	5	1.13	2	4.5	5	5.0	5
		#2	7	4.57	5	0.79	3	4.5	5	5.0	5
		#3	7	4.29	5	1.11	2	4.0	5	5.0	5
		#4	7	4.71	5	0.49	4	4.5	5	5.0	5
		#5	7	4.43	5	1.13	2	4.5	5	5.0	5
	Combined	#1	21	4.29	5	1.15	1	4.0	5	5.0	5
		#2	21	4.33	5	0.91	2	4.0	5	5.0	5
		#3	21	4.33	5	1.11	2	4.0	5	5.0	5
		#4	21	4.52	5	0.75	3	4.0	5	5.0	5
		#5	21	4.48	5	0.98	2	4.0	5	5.0	5

Readers used a 5-point Likert scale (1 = strongly agree; 2 = agree; 3 = neutral; 4 = disagree; and 5 = strongly disagree)

*IQR* Interquartile range, *Max* Maximum, *Min* Minimum, *Q1* First quartile, *Q3* Third quartile, *SD* Standard deviation

#### Mammography reports

For factual accuracy and completeness, scores ranged from 1 to 4 and the median score was 2 (IQR 2) for all five BRRs. For potential harm, scores ranged from 3 to 5 and the median score was 5 (IQR 5) for all five readers.

#### Ultrasound reports

For factual accuracy, scores ranged from 1 to 4 and the median score was 1 (IQR 1) for BRRs #1 and #2 while it was 2 (IQR 2) for BRRs #3, #4, and #5. For completeness, scores ranged from 1 to 3 and the median score was 1 (IQR 1) for BRRs #1, #2, and #5 while it was 2 for BRRs #3 and #4. For potential harm, scores ranged from 1 to 5 and the median score was 4 (IQR 4) for BRRs #1 and #2 while it was 5 for BRRs #3, #4, and #5.

#### MRI reports

For factual accuracy, scores ranged from 1 to 3 and the median score was 1 (IQR 1) for BRR #4 and 2 (IQR 2) for BRRs #1, #2, #3, and #5. For completeness, scores ranged from 1 to 3 and the median score was 1 (IQR 1) for BRRs #1 and #3 and 2 for BRRs #2, #4, and #5. For potential harm, scores ranged from 2 to 5 and the median score was 5 (IQR 5) for all the BRRs.

#### Combined analysis

For factual accuracy, scores ranged from 1 to 4 and the median score was 2 (IQR 2) were 2. For completeness, scores ranged from 1 to 4 and the median score was 1 (IQR 1) for BRR #1 and 2 (IQR 2) for BRRs #2, #3, #4, and #5. For potential harm, scores ranged from 1 to 5 and the median score was 5 (IQR 5) for all BRRs.

#### Reliability of measures

Cronbach’s α was 0.863 for factual accuracy, 0.884 for completeness, and 0.922 for potential harm. The Kruskal–Wallis test revealed no significant differences among the BRRs’ ratings within each modality and quality aspects, with the following results: mammography, factual accuracy (*p* = 0.962), completeness (*p* = 0.976), and potential harm (*p* = 0.975); ultrasound, factual accuracy (*p* = 0.980), completeness (*p* = 0.829), and potential harm (*p* = 0.780); and MRI, factual accuracy (*p* = 0.854), completeness (*p* = 0.880), and potential harm (*p* = 0.969).

#### Differences between the BI-RADS categories

For completeness, the Mann–Whitney *U*-test revealed a significant difference between the BI-RADS 0, 1, and 2 groups and the BI-RADS 3, 4, 5, and 6 groups (*p* = 0.001). Regarding factual accuracy and potential harm, the test did not reveal any statistically significant differences (*p* = 0.254 and *p* = 0.778, respectively). For all the categories the Mann–Whitney *U*-test revealed a significant difference between the BI-RADS 3 group and the BI-RADS 4 and 5 group (*p* < 0.001).

### NHRs’ assessments

#### Descriptive analysis of the NHRs’ comprehension ratings for mammography, ultrasound, and MRI reports

**Fig. 5:**
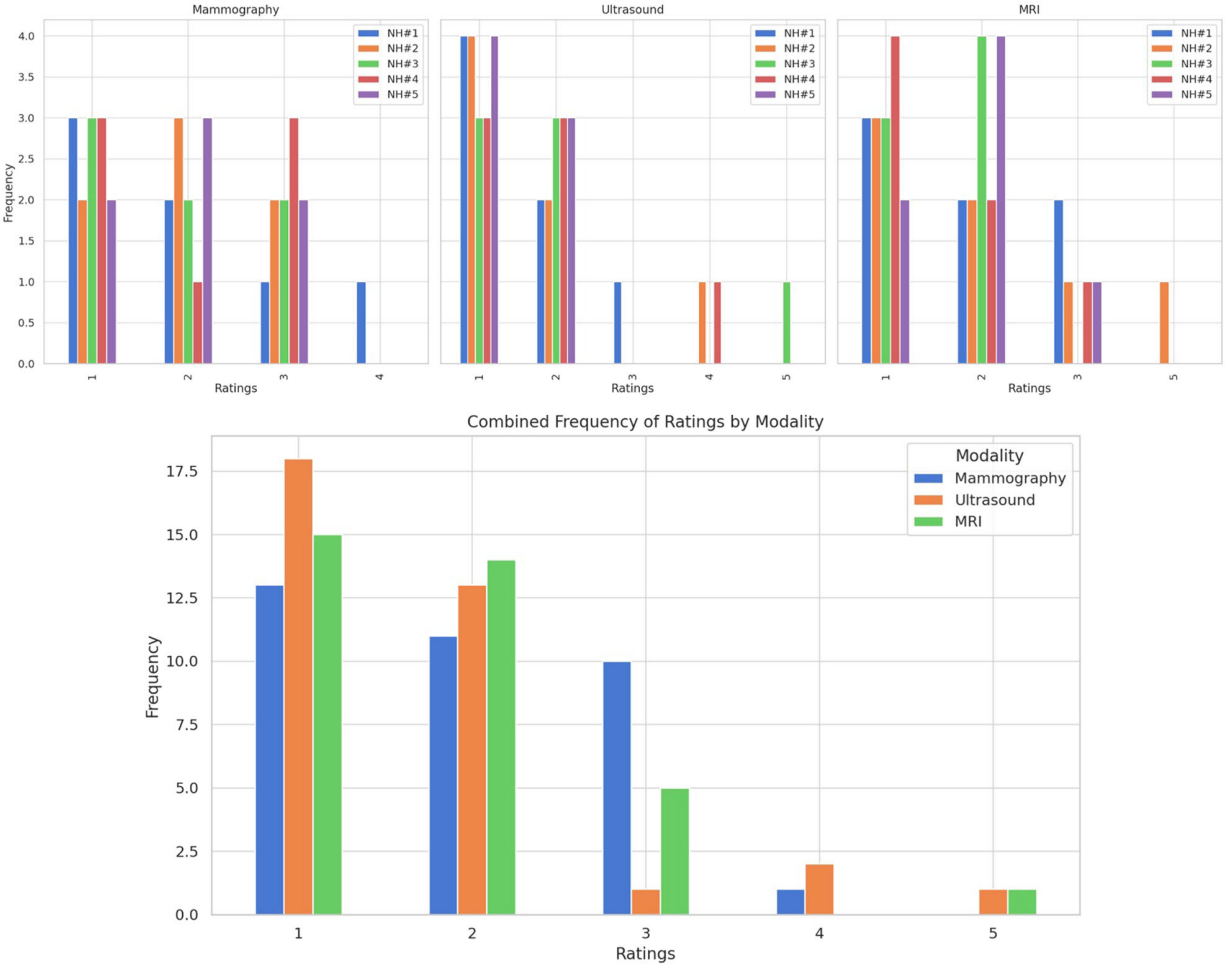
The frequency of NHRs’ understanding ratings for mammography, ultrasound, and MRI reports (on the top), as well as the combined frequency (on the bottom), using a 5-point Likert scale (1 = excellent; 2 = good; 3 = fair; 4 = adequate; and 5 = poor)

The results for each NHR understanding ratings across the three different imaging modalities, including the combined analysis, are detailed in Table 5. Frequency graphs of the NHR's understanding scores are presented in Fig. 5.

**Table 5:** Summary statistics for NHRs’ understanding

Modality	Reader	Mean	Median	SD	Minimum	Q1	IQR	Q3	Maximum
Mammography	NH#1	2.00	2.00	1.15	1.00	1.00	1.50	2.50	4.00
	NH#2	2.00	2.00	0.82	1.00	1.50	1.00	2.50	3.00
	NH#3	1.86	2.00	0.90	1.00	1.00	1.50	2.50	3.00
	NH#4	2.00	2.00	1.00	1.00	1.00	2.00	3.00	3.00
	NH#5	2.00	2.00	0.82	1.00	1.50	1.00	2.50	3.00
	Combined	1.97	2.00	0.94	1.00	1.00	2.00	3.00	4.00
Ultrasound	NH#1	1.57	1.00	0.79	1.00	1.00	1.50	2.50	3.00
	NH#2	1.71	1.00	1.11	1.00	1.00	2.00	3.00	4.00
	NH#3	2.00	2.00	1.41	1.00	1.00	2.50	3.50	5.00
	NH#4	1.86	2.00	1.07	1.00	1.00	2.00	3.00	4.00
	NH#5	1.43	1.00	0.53	1.00	1.00	1.00	2.00	2.00
	Combined	1.71	1.00	0.98	1.00	1.00	1.00	2.00	5.00
MRI	NH#1	1.86	2.00	0.90	1.00	1.00	2.00	3.00	3.00
	NH#2	2.14	2.00	1.46	1.00	1.00	3.00	4.00	5.00
	NH#3	1.57	2.00	0.53	1.00	1.00	1.00	2.00	2.00
	NH#4	1.57	1.00	0.79	1.00	1.00	1.50	2.50	3.00
	NH#5	1.86	2.00	0.69	1.00	1.00	1.50	2.50	3.00
	Combined	1.81	2.00	0.88	1.00	1.00	1.00	2.00	5.00

Readers’ text comprehension was evaluated by a 5-point Likert scale (1 = excellent; 2 = good; 3 = fair; 4 = adequate; and 5 = poor)

*IQR* Interquartile range, *Q1* First quartile, *Q3* Third quartile, *SD* Standard deviation

#### Mammography reports

For mammography, the median ratings for all five readers were consistently 2.

IQR were 1 for NHR #2 and #5, 1.5 for NH R#1, and #3 and 2 for NHR #4.

#### Ultrasound reports

In the case of ultrasound, the median ratings were 1 for NHR#1, #2, and #5, and 2.00 for NHR#3 and #4. IQR were 1 for NHR#5, 1.5 for NHR#1, 2 for NHR#2 and NHR#4, and 2.5 for NHR#3.

#### MRI reports

For MRI, the median ratings were 2 for NHR#1, #2, #3, and #5, while NHR#4 had a median rating of 1.00. IQR were 1 for NHR#3, 1.5 for NHR#4 and #5, 2 for NHR#1, and 3 for NHR#2.

#### Reliability of measures

Cronbach’s α was 0.968 for mammography, 0.856 for ultrasound, and 0.890 for MRI, indicating a high level of internal consistency among the ratings for each modality. The nonparametric Kruskal–Wallis test revealed no statistically significant differences in the comprehension ratings between the different imaging modalities (*p* = 0.368).

#### Differences between the BI-RADS categories

The Mann–Whitney *U*-test did not reveal a significant difference between the BI-RADS 0, 1, and 2 groups and the BI-RADS 3, 4, 5, and 6 groups (*p* = 0.282). The Mann–Whitney *U*-test revealed a significant difference between the BI-RADS 3 group and the BI-RADS 4 and 5 group (*p* < 0.001).

## Discussion

The terminology used in radiological reports is often specialized and complex, requiring specific experience and training to understand [10]. In our experience, patients often need a doctor’s help to fully comprehend these reports. This issue is common with breast radiology reports, which can be confusing due to frequent abbreviations and classifications that are not familiar to most readers [11, 12]. Although doctors explain these investigations, patients are usually interested in reading their reports [13]. They may try to interpret the information on their own before consulting a healthcare professional, which can lead to misunderstandings and anxiety [13]. This problem affects many people who do not have a medical background. In a world increasingly focused on patient-centered care, it is crucial to address this issue. Making radiological reports clearer and more accessible can help patients understand their health better and be more involved in their care, leading to improved outcomes.

Patients often turn to the internet for information [14]. While the internet is a powerful resource, it has not effectively simplified medical texts like radiological reports, except through communication applications that connect patients with professionals [15]. However, the advent of LLMs like ChatGPT offers new possibilities. These AI-based tools, designed with a focus on language, can simplify complex texts. This innovation provides a promising solution for making medical information more accessible to patients, enhancing their understanding and engagement in their own healthcare.

The importance of this study lies in addressing a fundamental need to make medical information accessible to all, regardless of lexical complexity. This approach can assist both radiologists and patients by utilizing AI to simplify reports, ultimately benefiting both parties.

We evaluated the efficacy of AI-driven simplification of breast radiology reports using ChatGPT-4o. BRRs rated the simplified reports high for factual accuracy, and completeness, and low for potential harm. The high internal consistency (α > 0.80) and the absence of significant differences in ratings among radiologists (*p* > 0.05) further support the reliability of these evaluations. The AI simplifications maintained accuracy and completeness while minimizing potential harm, suggesting they can enhance patient understanding without significant risks.

According to the descriptive data analysis, the BI-RADS classification system appears to have significantly influenced the simplification process of the AI-simplified reports, however, a statistically significant difference was observed only for the completeness category (*p* = 0.001). Reports categorized as BI-RADS 0, 1, and 2, involving incomplete assessments or benign findings, were generally simpler to explain and received higher scores for factual accuracy and completeness. In contrast, BI-RADS 3, 4, 5, and 6 reports, which involve more complex and clinically significant findings, posed greater challenges for simplification. These reports required conveying nuanced or urgent information accurately without causing misunderstandings, which might explain the slightly lower scores in these categories. Overall, the AI performed better with simpler, less critical findings, while maintaining an acceptable level of accuracy and completeness with more complex cases.

However, it is important to note that while the AI-simplified reports generally demonstrated high factual accuracy and completeness, there were instances where the potential for harm was identified, particularly in more complex BI-RADS 3–6 cases, indicating the need for careful oversight and further refinement of these AI tools to ensure patient safety.

In the group of reports involving potentially malignant findings, namely BI-RADS 3, 4, and 5, it was found that BI-RADS 3 reports were more difficult to simplify compared to BI-RADS 4 and 5, with statistically significant differences (*p* < 0.001) for all the categories, highlighting a certain difficulty on the part of AI in explaining findings of questionable significance.

The neutral score of 3 in the ‘potential harm’ category suggests that while the AI-simplified reports were generally accurate and comprehensible, there were instances where the potential for patient misunderstanding was neither completely mitigated nor exacerbated, highlighting the need for ongoing refinement to ensure absolute clarity and safety.

NHRs showed a good level of comprehension of AI-simplified radiology reports across mammography, ultrasound, and MRI modalities. The high internal consistency (α > 0.80) suggests that the AI-generated simplified reports were reliably understood by the readers.

As observed in the evaluation of BRRs, the descriptive analysis revealed that although no statistically significant differences were found (*p* = 0.282), the comprehension of NHRs was similarly affected by the BI-RADS classification of the report. Specifically, BI-RADS categories 3, 4, 5, and 6 presented greater challenges for these readers, with BI-RADS 3 being particularly difficult to simplify (*p* < 0.001).

Our study aligns with findings from existing literature, reinforcing the potential of AI in medical text simplification. Jeblick et al [7] explored patients autonomously using ChatGPT to simplify their medical reports, finding that while the simplified reports were generally accurate, they could contain notable errors and potentially harmful passages. This supports our findings that, although ChatGPT-4o performs well in simplifying reports, complex cases require careful oversight to prevent misinterpretation. Both studies highlight the significant potential of ChatGPT-like models in medical text simplification, tempered by the necessity for professional supervision and ongoing refinement.

Similarly, Mallio et al [3] compared the performance of four LLMs (GPT-4, ChatGPT-3.5, Perplexity, and Bing) in generating structured radiology reports, noting variability in detail and presentation. Our study, focusing solely on ChatGPT-4o, confirms that a single well-tuned model can achieve high factual accuracy and completeness. However, our study stands out by emphasizing patient comprehension through simplified reporting, demonstrating the practical utility of AI in improving patient-centered care.

Our study aligns with Ali et al [5], who explored the performance of ChatGPT on a variety of dental education assessments. Both studies demonstrate the high accuracy and potential utility of ChatGPT in educational contexts. While Ali et al found that ChatGPT performed well across multiple question formats but struggled with image-based questions, our study similarly highlights the model’s strengths in textual comprehension and simplification but underscores the need for careful oversight in complex medical contexts. Both studies underscore the transformative potential of AI in education and healthcare, balanced by the necessity for adaptation and supervision to mitigate risks.

Collectively, the literature illustrates a consistent recognition of the potential benefits and limitations of using LLMs like ChatGPT in handling medical reporting; simplifying radiology reports through AI can enhance patient understanding, facilitate more effective communication, and strengthen patient-centered care.

Although promising, the use of LLMs such as ChatGPT-4 in clinical practice is not currently certified. The recent AI Act by the European Union Parliament imposes specific requirements for generative AI in high-risk applications, including healthcare, thereby rendering any use of generative AI in clinical practice unauthorized [16].

In a possible future, the integration of better AI tools into clinical practice could make radiological information more accessible to patients with varying educational backgrounds, thereby contributing to greater patient engagement and satisfaction. However, given that LLMs are accessible to anyone with internet access, it remains essential to evaluate their reliability, as they could potentially mislead patients.

Of note, this study, along with the LLM in general, does not fully address the significant ethical issues inherent in the doctor-patient relationship. However, we believe that despite these ethical concerns, AI models can be beneficial and useful in circumstances where access to medical opinions is limited or unavailable.

This study has several limitations: the use of a small number of randomly selected radiology reports, the reliance on a single AI model (ChatGPT-4o), and the potential evaluator bias (the variability in AI outputs and the BRRs’ familiarity with the BI-RADS classification may also influence findings). Future research should use customizable AI settings and a diverse assessment team.

In conclusion, our study shows that ChatGPT-4 can effectively simplify breast radiology reports, making them more accessible to patients without losing key information. Simplifications worked best for simpler cases, like BI-RADS 0–2, while more complex cases require oversight to prevent errors. Although NHRs understood the simplified reports, further refinement of AI tools is needed to ensure patient safety, especially for more nuanced findings. Additionally, given the easy access to AI online, this study is also important to assess the reliability of LLMs for patients using them at home without medical supervision.

Future research should build on these findings exploring customizable AI settings to further validate and refine the application of AI in radiological reporting.

## Data Availability

Data will be made available on reasonable request.

## Abbreviations

AI: Artificial intelligence
BI-RADS: Breast imaging reporting and data system
BRR: Breast radiologist reader
IQR: Interquartile range
LLMs: Large language models
MRI: Magnetic resonance imaging
NHR: Non-healthcare reader
